# Evaluating *Musca domestica* as an alternative to *Lucilia sericata* in maggot-assisted wound healing: A comparative study of therapeutic efficacy in a murine model

**DOI:** 10.1016/j.parepi.2026.e00517

**Published:** 2026-05-28

**Authors:** Kourosh Azizi, Vahid Saleh, Qasem Asgari, Akbar Safaei, Sorna Dabaghmanesh, Mohsen Kalantari

**Affiliations:** a Research Center for Health Sciences, Institute of Health, Department of Biology and Control of Disease Vectors, School of Health, Shiraz University of Medical Science, Shiraz, Iran; b Student Research Committee, Department of Biology and Control of Disease Vectors, School of Health, Shiraz University of Medical Sciences, Shiraz, Iran; c Department of Parasitology and Mycology, School of Medicine, Shiraz University of Medical Sciences, Shiraz, Iran; d Department of Pathology, Shiraz University of Medical Sciences, Shiraz, Iran

**Keywords:** Maggot debridement therapy, *Musca domestica*, *Lucilia sericata*, *Pseudomonas aeruginosa*, Burn wound infection, Biotherapy, Antibiotic resistance

## Abstract

The emergence of antibiotic-resistant infections has renewed interest in Maggot Debridement Therapy (MDT) as a potent biotherapy for wound care. While *Lucilia sericata* is the gold standard species, its specialized rearing requirements and associated costs can be a barrier to implementation in resource-limited settings. This study evaluated the common housefly, *Musca domestica*, as a potential alternative for treating *Pseudomonas aeruginosa*-infected burn wounds in a rat model. In this study, seventy rats were assigned to seven groups: Control (C), Infected control (B), Antibiotic only (BA), *L. sericata* only (BL), *L. sericata* + Antibiotic (BLA), *M. domestica* only (BM), and *M. domestica* + Antibiotic (BMA). Wound area, bacterial load, and histological parameters were assessed over 22 days. Life tables were constructed for both fly species under laboratory conditions. Results showed that both larval species significantly enhanced wound healing compared to infected controls (*p* < 0.05). *L. sericata* groups (BL, BLA) exhibited the most rapid healing, with complete wound closure observed in some subjects. The *M. domestica* groups (BM, BMA) also demonstrated significant debridement and healing, though at a slightly slower rate. The combination of larvae with antibiotics yielded the most effective reduction in bacterial load. Histological analysis revealed enhanced granulation tissue formation and epithelialization in larva-treated groups. The net reproductive rate (R0) was higher for *L. sericata* (3.398) than for *M. domestica* (2.206). We conclude that while *L. sericata* remains more efficacious, *M. domestica* presents a viable, accessible, and effective alternative for MDT, particularly in resource-limited settings, offering a promising strategy to combat antibiotic-resistant wound infections.

## 1 Introduction

Maggot Debridement Therapy (MDT), also known as larval therapy, is an ancient yet innovative approach to wound care that has been utilized for centuries across various cultures (Simonetti et al., 2025). Its historical use was documented in traditional medicine, where maggots were applied to necrotic or infected wounds to promote healing (Mortlock, 2024). In modern medicine, MDT has re-emerged as a scientifically validated treatment, particularly for chronic and antibiotic-resistant wounds (Bhadoriya et al., 2023). The therapy involves the controlled application of sterile fly larvae to debride necrotic tissue, disinfect the wound, and stimulate tissue regeneration (Kenawy and Abdel-Hamid, 2020). With the escalating global challenge of antimicrobial resistance (AMR), MDT offers a promising alternative to conventional antibiotic treatments, especially for wounds infected with multidrug-resistant pathogens such as *Pseudomonas aeruginosa* (Puri et al., 2025).

The primary mechanism of MDT involves three synergistic actions: debridement, disinfection, and wound healing stimulation (Gao et al., 2025). The larvae secrete proteolytic enzymes that liquefy and consume necrotic tissue while sparing healthy tissue, a process known as selective debridement (Nigam and Wilson, 2022). Additionally, maggots produce antimicrobial peptides and other bioactive molecules that inhibit bacterial growth, including pathogens like *P. aeruginosa* and methicillin-resistant *Staphylococcus aureus* (MRSA) (Owusu and Redfern, 2025). These antimicrobial properties are particularly valuable in managing biofilms, which are notoriously resistant to antibiotics (Tuon et al., 2023). Furthermore, larval secretions contain growth factors such as transforming growth factor-β (TGF-β) and vascular endothelial growth factor (VEGF), which enhance angiogenesis, granulation tissue formation, and epithelialization, thereby accelerating wound closure (Shanmugam et al., 2022).

The green bottle blowfly, *Lucilia sericata* (Diptera: Calliphoridae), is the most widely studied and utilized species in MDT due to its efficacy in wound debridement and infection control (Bazaliński et al., 2023). Its larvae are highly specialized for necrophagy, making them ideal for clinical applications (Moemenbellah-Fard et al., 2024). However, the establishment of standardized, sterile rearing facilities for *L. sericata*, while feasible, requires significant infrastructure, technical expertise, and cost, which can be prohibitive in many resource-limited regions (Bazaliński et al., 2023). This practical and economic barrier necessitates the exploration of alternative, more readily cultivable species. In contrast, the housefly, *Musca domestica* (Diptera: Muscidae), is ubiquitous, highly adaptable, and can be reared efficiently with simpler protocols and at a substantially lower cost (Geden et al., 2021). Its potential for decentralized, local production makes it a compelling candidate for expanding MDT access. Despite its reputation as a mechanical vector for pathogens, *M. domestica* exhibits significant potential for MDT, an aspect that has received limited attention in the scientific literature (Ali et al., 2024).

The rising prevalence of chronic wounds, such as diabetic foot ulcers, venous leg ulcers, and burn injuries, underscores the urgent need for effective and cost-efficient therapies (Sabarees et al., 2025). Current treatments often involve surgical debridement, antibiotics, and advanced wound dressings, which can be invasive, expensive, or ineffective against resistant infections (Cristea et al., 2025). MDT presents a minimally invasive, cost-effective alternative with a high success rate in clinical settings (Mumford et al., 2024). For instance, studies have demonstrated that MDT can reduce the need for amputations in diabetic patients with non-healing ulcers and improve outcomes in burn wounds infected with *P. aeruginosa* (Cutteridge and Bera, 2021). Moreover, the therapy is associated with fewer side effects compared to systemic antibiotics, which may cause adverse reactions or disrupt the microbiome (Lam et al., 2025).

Despite its advantages, MDT faces challenges in widespread adoption, including patient aversion, regulatory hurdles, and the need for standardized protocols (Redford et al., 2024). This study aims to address the accessibility issue by evaluating the common housefly, *Musca domestica*, as a potential alternative to the established *Lucilia sericata*. We hypothesize that *M. domestica* larvae possess significant debridement and antimicrobial capabilities that can be harnessed for therapeutic purposes. By examining wound healing rates, bacterial load reduction, and histological outcomes in a controlled murine model of *P. aeruginosa*-infected burn wounds, this research seeks to determine the comparative efficacy of these two species and explore the potential for a combined approach with antibiotics.

The findings of this study could have significant implications for wound care, particularly in resource-limited settings where *L. sericata* may not be readily available. If effective, *M. domestica* could expand the accessibility of MDT, offering a scalable solution for managing chronic and infected wounds. Furthermore, this research contributes to the growing body of evidence supporting biotherapy as a sustainable and innovative approach to combating antibiotic resistance.

## 2 Materials and methods

### 2.1 Study design and ethical considerations

This experimental study was designed to evaluate and compare the efficacy of *Musca domestica* larvae to the gold-standard *Lucilia sericata* in Maggot Debridement Therapy (MDT) for treating *Pseudomonas aeruginosa*-infected burn wounds in an albino rat model. The study protocol was approved by the Ethics Committee of Shiraz University of Medical Sciences (Ethics No: IR.SUMS.REC.1390.S6129) and adhered to national guidelines for the humane treatment of laboratory animals. All procedures were conducted under sterile conditions to minimize contamination and ensure reproducibility.

### 2.2 Fly collection and rearing

#### 2.2.1 Collection of adult flies

Adult *Lucilia sericata* specimens were collected from the Shiraz Central Slaughterhouse using insect nets, where they were attracted to decomposing organic matter. *Musca domestica* adults were captured from a poultry farm in Shiraz, Iran, using baited traps. Taxonomic identification was performed for 5% of the collected specimens using the Pictorial key (National Communicable Disease Center (US), 1969). The remaining 95% were transferred to a controlled insectarium for mass breeding.

#### 2.2.2 Insectarium conditions

Both species were reared in a climate-controlled insectarium maintained at 26–28 °C, with relative humidity levels between 40% and 60%, and a 12:12-h light-dark cycle. Adult flies were housed in mesh cages (45 × 45 × 45 cm). Water was provided ad libitum via a cotton-plugged vial containing a 3% sucrose solution for both species.

##### 2.2.2.1 Rearing of *Lucilia sericata*

Adult *L. sericata* were provided with a diet consisting of a mixture of 50% MacConkey agar powder and 50% sucrose, moistened with water. For oviposition, fresh, scarified sheep liver was provided. Eggs were collected within 24 h and sterilized for subsequent use in MDT.

##### 2.2.2.2 Rearing of *Musca domestica*

Adult *M. domestica* were provided with a diet consisting of a mixture of 50% MacConkey agar powder and 50% sucrose, moistened with water, alongside apple slices which also served as an oviposition substrate. Eggs were collected from the apple slices within 24 h and sterilized for subsequent use in MDT.

### 2.3 Sterilization and preparation of larvae

#### 2.3.1 Egg sterilization protocol

To ensure aseptic conditions for wound application, the following sterilization steps were implemented:

2.3.1.1. **Initial Screening:** Healthy eggs were separated from damaged ones by submerging them in distilled water; floating eggs were discarded.
2.3.1.2. **Chemical Sterilization:** Eggs were agitated in a 0.25% chloramine-T solution for three intervals of 20 min each using an orbital shaker (100 rpm).
2.3.1.3. **Rinsing and Storage:** After sterilization, eggs were rinsed three times with sterile distilled water and stored in an air-filtered flask at 25 °C until hatching.

#### 2.3.2 Larval rearing medium

A nutrient-rich culture medium was prepared by dissolving 1 mL of BHI (Brain Heart Infusion) broth and 1 mL of standard agar in a bain-marie. Eggs were transferred to this medium and incubated at 26 °C. Hatched larvae (first instar) were inspected for sterility after 24 h; contaminated batches were discarded. First-instar larvae from sterile batches were used for all MDT applications.

### 2.4 Life table construction

To assess the reproductive potential and evaluate the practical feasibility of mass rearing for MDT programs, life tables were constructed for both species. For each species, 100 newly emerged adults (with a 1:4 male-to-female ratios for *L. sericata* and a 1:1 ratio for *M. domestica*) were placed in separate cages. The number of eggs laid, hatched larvae, developed pupae, and emerged adults was recorded daily for three consecutive generations (F1-F3) (Firoozfar et al., 2012). Key parameters including survival rate (lₓ), mortality rate (dₓ), net reproductive rate (R₀), and mean generation time were calculated according to standard entomological methods (Southwood and Henderson, 2009).

### 2.5 Animal model preparation

Animal ethic notes All animal experimental protocols were approved by the Science and Ethics Committee of Shiraz University of Medical Sciences.

#### 2.5.1 Experimental animals

Seventy adults female *Rattus norvegicus* (Westar albino strain), weighing 180–250 g, were obtained from the Comparative and Experimental Medicine Center at Shiraz University of Medical Sciences. Rats were acclimatized for one week in individual cages under controlled conditions (22 ± 2 °C, 12:12 light-dark cycle) with ad libitum access to water and standard rodent chow.

#### 2.5.2 Burn wound induction

**Fig. 1:**
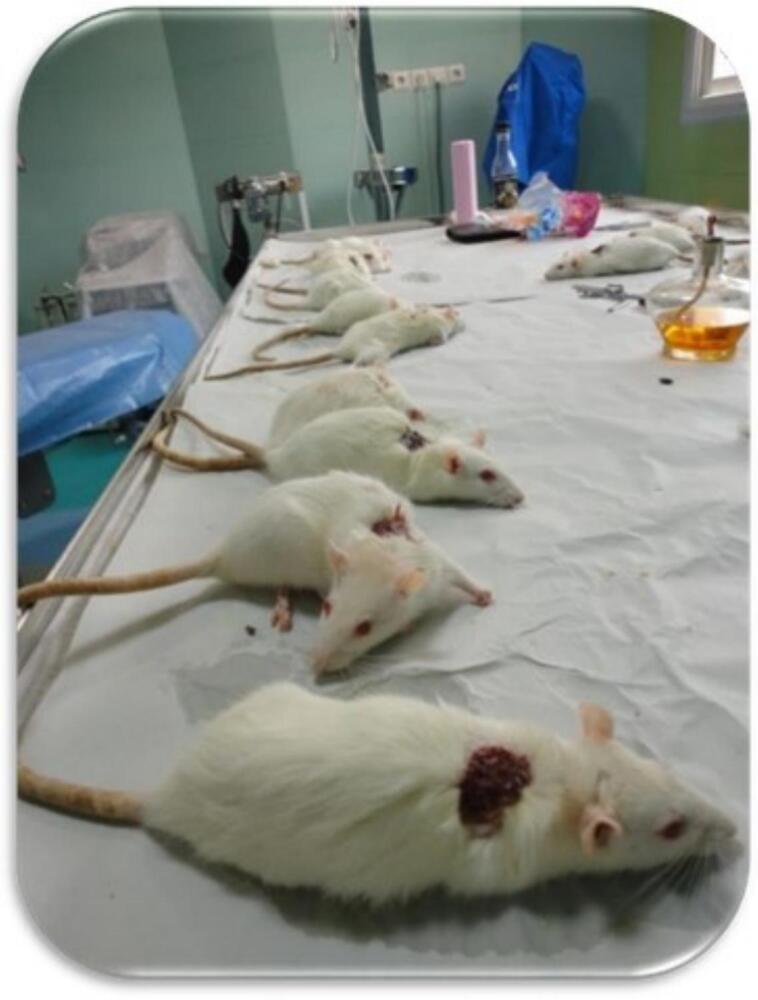
Inducing burns on animal models.

• **Anesthesia:** Rats were anesthetized via intraperitoneal injection of a ketamine (80 mg/kg) and diazepam (5 mg/kg) cocktail.
• **Hair Removal and Disinfection:** The dorsal surface was shaved and disinfected with 5% povidone‑iodine solution.
• **Burn Creation:** A preheated 2 × 2 cm metal plate (100 °C) was applied to the skin for 5 s to induce a standardized second-degree burn (epidermis and superficial dermis damage without deeper tissue involvement) (Fig. 1).

#### 2.5.3 Wound infection with *Pseudomonas aeruginosa*

**Fig. 2:**
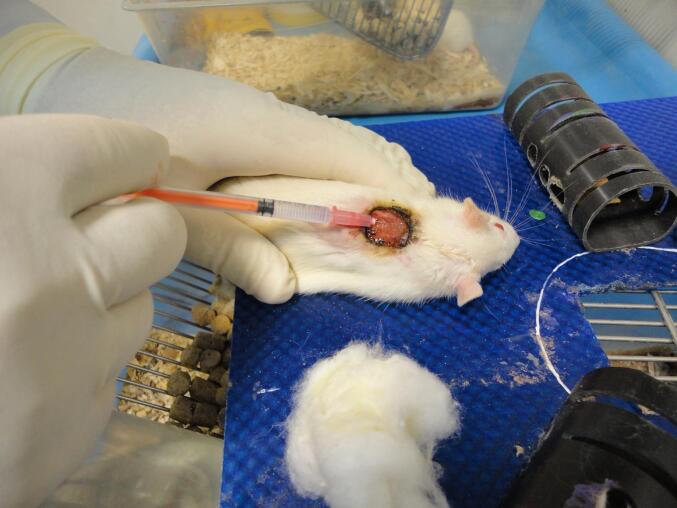
Inoculation of the burn wound with *Pseudomonas aeruginosa***.**

After 48 h, the burn eschar was mechanically removed, and a clinical strain of *P. aeruginosa* isolated from a burn patient (1.0 McFarland standard, ∼3 × 10^8^ CFU/mL) was inoculated subcutaneously into the wound bed using a sterile insulin syringe (1 mL volume) (Fig. 2). The wound was covered with sterile gauze and secured with adhesive tape to facilitate infection. Infection was confirmed 72 h post-inoculation by bacterial culture before commencing treatments.

### 2.6 Experimental groups and treatment protocols rats were randomly allocated into seven groups (*n* = 10 per group)

1. **Control (C):** Non-infected burn wounds, no treatment.
2. **B (Infected Control):** *P. aeruginosa*-infected wounds, no therapy.
3. **BA (Antibiotic Only):** Infected wounds treated with ceftriaxone (50 mg/kg/day, IM) for 20 days.
4. **BL (*L. sericata* Only):** Infected wounds treated with 16–20 sterile first-instar *L. sericata* larvae per wound.
5. **BLA (*L. sericata* + Antibiotic):** Combination therapy with larvae (as in BL) and ceftriaxone (as in BA).
6. **BM (*M. domestica* Only):** Infected wounds treated with 12–16 sterile first-instar *M. domestica* larvae per wound.
7. **BMA (*M. domestica* + Antibiotic):** Combination therapy with larvae (as in BM) and ceftriaxone (as in BA).

### 2.7 Maggot therapy application (Fig. 3)

• Larvae were applied every other day for four sessions, each lasting 3 h, under anesthesia.
• Wounds were dressed with sterile, moist gauze to maintain humidity and prevent larval escape.
• Antibiotic-treated groups received ceftriaxone concurrently.

**Fig. 3:**
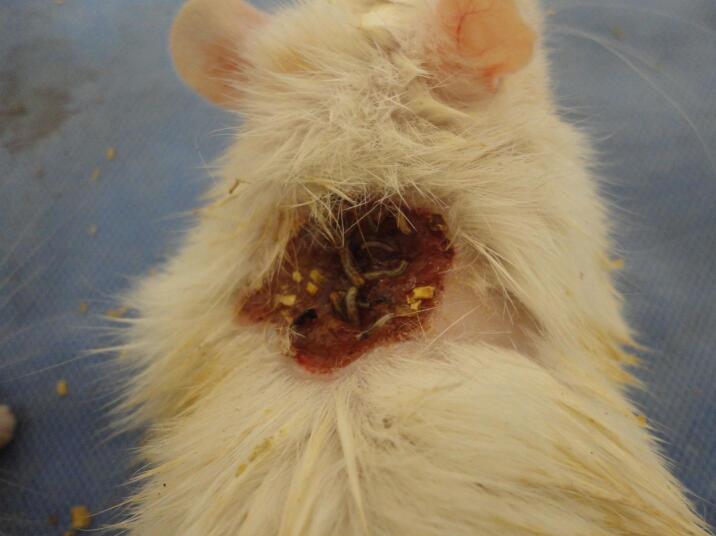
Applying the larvae in the wounds of Balb/c.

### 2.8 Wound assessment and data collection

#### 2.8.1 Healing evaluation

Wound dimensions (length, width) were measured on days 0, 3, 6, 9, 12, 15, 18, and 22 post-infections using a digital Vernier caliper (0.01 mm precision). The wound area was calculated assuming an elliptical shape: Area = (Length × Width × π) / 4. The size of wound healing was calculated as: [(Initial Area - Final Area) / Initial Area].

#### 2.8.2 Microbiological analysis

On days 7, 14, and 22, wound swabs were collected aseptically for bacterial culture on MacConkey and blood agar plates. Colony-forming units (CFUs) of *P. aeruginosa* were quantified after 24 h incubation at 37 °C.

#### 2.8.3 Histopathological examination

At the endpoint (day 22), euthanized rats underwent punch biopsies (5 mm) from the wound site. Tissues were fixed in 10% formalin, processed, and stained with Hematoxylin & Eosin (H&E) and Masson's Trichrome. A blinded pathologist assessed:

• Granulation tissue formation.
• Collagen deposition.
• Inflammatory cell infiltration.
• Epithelialization (Epidermalization).

### 2.9 Statistical analysis

Data were analyzed using SPSS v26.0. Normality was assessed using the Shapiro-Wilk test. One-way ANOVA followed by Tukey's post hoc test was used to compare healing rates and CFU counts between groups. CFU counts were log10-transformed to achieve normality. Histological scores were compared using the Kruskal-Wallis test. Statistical significance was set at *p* < 0.05. Data are presented as mean ± standard deviation (SD).

## 3 Results

### 3.1 Life table analysis

The life table parameters for both fly species reared under laboratory conditions are summarized in Table 1. To assess their potential for sustainable mass rearing—a key factor for clinical accessibility**—** we calculated the net reproductive rate (R₀). *Lucilia sericata* exhibited a higher net reproductive rate (R₀ = 3.398) compared to *Musca domestica* (R₀ = 2.206). The total development time (egg to adult) was shorter for *L. sericata* (approximately 9 days) than for *M. domestica* (approximately 11.7 days). The survival rate from egg to adult was 73.8% for *L. sericata* and 70.9% for *M. domestica*. Mortality was highest during the larval stages for *M. domestica* (qₓ = 0.097, 0.076, 0.093 for L1, L2, L3, respectively), whereas for *L. sericata*, the highest mortality occurred at the egg stage (qₓ = 0.125).

**Table 1:** Comparative life table parameters for *Lucilia sericata* and *Musca domestica* under laboratory conditions.

Parameter	*L. sericata*	*M. domestica*
Net Reproductive Rate (R₀)	3.398	2.206
Mean Generation Time (days)		
Survival Rate (egg to adult, %)	73.8	70.9
Mortality Rate (qₓ) - Egg	0.125	0.038
Mortality Rate (qₓ) - Larvae I	0.067	0.097
Mortality Rate (qₓ) - Larvae II	0.026	0.076
Mortality Rate (qₓ) - Larvae III	0.059	0.093
Mortality Rate (qₓ) - Pupae	0.013	0.026

### 3.2 Wound healing progression

**Fig. 4:**
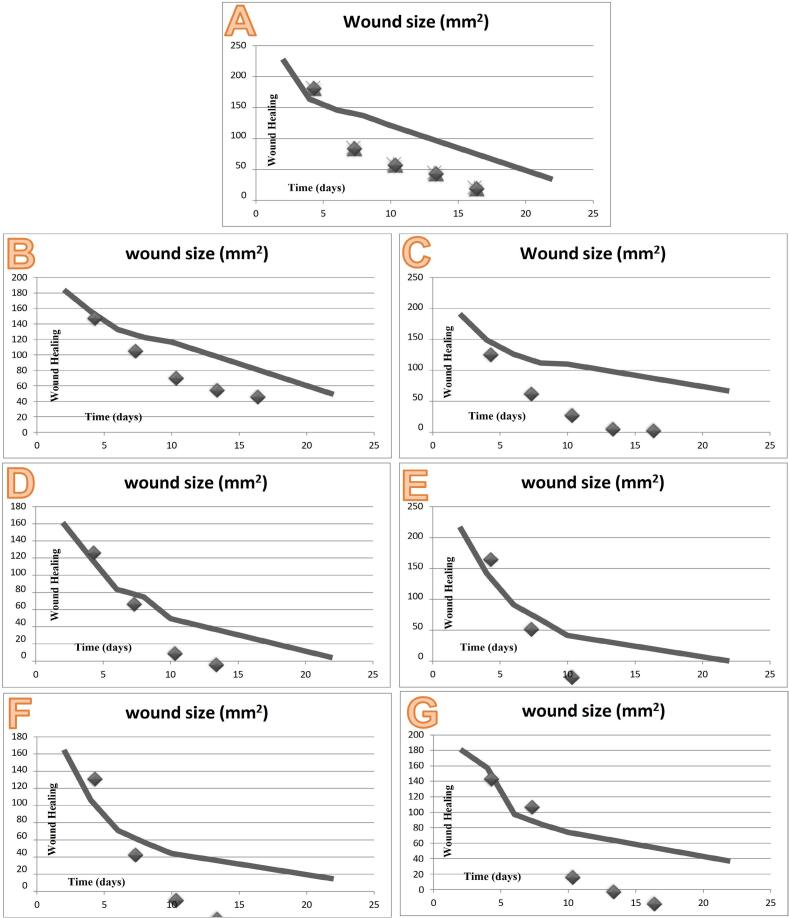
Wound healing over time in different experimental groups. The x-axis represents Time (days) and the y-axis represents the Wound Healing size (mm^2^). (A: Control (C), B: Infected Control (B), C: Antibiotic Only (BA), D: *L. sericata* Only (BL), E: *L. sericata* + Antibiotic (BLA), F: *M. domestica* Only (BM), G: *M. domestica* + Antibiotic (BMA).

All treatment groups showed significant improvement in wound healing compared to the infected control group (B) (*p* < 0.001). The progression of wound closure over time for all groups. The most rapid healing was observed in groups treated with *L. sericata* larvae (BL and BLA). By day 22, wounds in the BLA group showed near-complete closure (97.5% healing), followed by the BL group (91.5% healing). Groups treated with *M. domestica* (BM and BMA) also demonstrated significant healing (79.6% and 85% respectively), outperforming the antibiotic-only group (BA, 73.4% healing) and the infected control group (B, 65.5% healing). The non-infected control group (C) healed at a rate of 100% by day 22. Statistical analysis (one-way ANOVA) confirmed that the differences in healing rates between the larva-treated groups and the infected control were highly significant (F = 31.842, *p* < 0.0001) (Fig. 4).

### 3.3 Microbiological analysis

Bacterial load, measured as CFU/swab of *P. aeruginosa*, decreased significantly in all treatment groups compared to the infected control (B) (*p* < 0.05). The most effective reduction was observed in the combination therapy groups (BLA and BMA), where CFUs were reduced to negligible levels by day 22. The *L. sericata* only group (BL) showed a more rapid and pronounced reduction in bacterial load compared to the *M. domestica* only group (BM). The antibiotic-only group (BA) showed a steady decline, but the presence of larvae accelerated this process. Wound swabs from the non-infected control (C) remained sterile throughout the study.

### 3.4 Histopathological findings

Histological examination revealed marked differences between groups. The infected control group (B) showed extensive inflammation, minimal granulation tissue, and no epithelialization. In contrast, larva-treated groups (BL, BLA, BM, BMA) exhibited robust formation of granulation tissue, organized collagen deposition, and significantly advanced epithelialization. Quantification of these parameters indicated that the scores for granulation tissue formation and epithelialization were 55% and 30% higher, respectively, in the BL group compared to the B group. The BM group also showed significant improvement, with scores 40% and 25% higher for granulation and epithelialization, respectively, compared to the infected control. The combination groups (BLA, BMA) showed the most organized tissue architecture, resembling the non-infected control (C).

## 4 Discussion

This study provides compelling evidence that Maggot Debridement Therapy (MDT) using both *Lucilia sericata* and *Musca domestica* larvae significantly enhances the healing of *Pseudomonas aeruginosa*-infected burn wounds in a rat model. The findings robustly confirm the established efficacy of *L. sericata* and, more importantly, demonstrate the considerable therapeutic potential of the common housefly, *M. domestica*, as a viable and accessible alternative for biotherapy, particularly in resource-limited settings (Ugoh and Dahunsi, 2024).

### 4.1 Reaffirming the gold standard: the efficacy of *L. sericata*

The superior performance of *L. sericata* observed in this study—evidenced by the most rapid wound closure, near-complete healing in combination groups, and a more pronounced reduction in bacterial load—aligns seamlessly with its well-documented biology as a specialist necrophagous species (Nigam et al., 2010). Its evolutionary adaptation to feed on necrotic tissue is facilitated by a potent cocktail of proteolytic enzymes such as collagenase and serine proteases, which efficiently liquefy and debride devitalized tissue while sparing healthy structures, a process known as selective debridement (Chambers et al., 2003). Furthermore, our life table analysis revealed a higher net reproductive rate (R₀ = 3.398) for *L. sericata* compared to *M. domestica*. Although this parameter does not directly relate to therapeutic action, this inherent prolificacy under controlled laboratory conditions is a significant logistical advantage for standardized mass rearing. This analysis was crucial for our objective of evaluating *M. domestica* as a practical alternative, as it quantifies rearing efficiency**.** While *L. sericata*'s inherent prolificacy is an advantage, the life table confirmed that *M. domestica* can also be reared efficiently (R₀ = 2.206) in a controlled setting. This reliable and sterile supply of larvae is a cornerstone for the widespread adoption of any biotherapy, and the data demonstrate that *M. domestica* is logistically feasible for mass production (Bazaliński et al., 2023).

The potent antimicrobial secretions of *L. sericata*, including defensin-like peptides and other antibacterial factors active against a broad spectrum of pathogens including MRSA and *P. aeruginosa*, underpin its powerful disinfecting action (Bexfield et al., 2004; Owusu and Redfern, 2025). This dual action of mechanical debridement and biochemical disinfection creates an optimal wound bed for healing.

### 4.2 The housefly as a novel therapeutic agent: unveiling *M. domestica*'s potential

The most significant contribution of this research is the empirical validation of *M. domestica* as an effective agent for MDT. While slightly less efficacious than *L. sericata*, the housefly larvae led to markedly better outcomes than antibiotic treatment alone or the infected control. This debridement effect is directly attributable to the larval feeding activity on necrotic tissue. Notably, the significant reduction in bacterial load in the BM group suggests that *M. domestica* larvae possess inherent, albeit less characterized, antimicrobial properties. This finding challenges the housefly's reputation solely as a disease vector and highlights its untapped therapeutic potential (Ali et al., 2024). The immune system of insects, including Diptera, produces a range of antimicrobial peptides (AMPs) in response to microbial challenge. It is plausible that *M. domestica* larvae secrete a unique profile of such molecules when introduced to an infected wound, contributing to bacterial clearance (Ugoh and Dahunsi, 2024). Further proteomic studies are warranted to isolate and characterize these specific bioactive compounds from *M. domestica* excretions/secretions (ES). The life table analysis, therefore, provides a foundational justification for its use beyond its therapeutic efficacy, by confirming that *M. domestica* can be reared efficiently (R₀ = 2.206) in a controlled setting. Its ubiquity, adaptability, and simpler dietary requirements, combined with this proven rearing efficiency, make it a highly practical and scalable candidate for MDT programs in regions where *L. sericata* is not endemic or its rearing is logistically challenging (Geden et al., 2021).

### 4.3 Synergistic interactions: MDT and antibiotic combination therapy

A key finding across both fly species was the superior outcome observed in the combination therapy groups (BLA and BMA), which exhibited the most effective bacterial clearance and the most organized tissue regeneration. This demonstrates a powerful synergy between larval therapy and systemic antibiotics (ceftriaxone). The mechanism for this synergy is likely multifaceted. Larvae physically disrupt bacterial biofilms—a major barrier to antibiotic efficacy in chronic wounds—through their movement and feeding activity (Tuon et al., 2023). By consuming the necrotic tissue that harbors embedded bacteria, larvae remove the physical sanctuary and nutrient source for pathogens like *P. aeruginosa* (Cowan et al., 2013). This debridement and biofilm disruption likely enhance vascular perfusion and improve the penetration and bioavailability of systemically administered antibiotics to the site of infection (Lam et al., 2025). This combined approach represents a promising, multi-pronged strategy to manage deep-seated, antibiotic-resistant infections, potentially reducing the required duration or dosage of antibiotics and mitigating the risk of further resistance development.

### 4.4 Histological corroboration of healing mechanisms

The histopathological results provide a cellular and molecular basis for the macroscopic healing observed. The enhanced granulation tissue formation, organized collagen deposition, and significantly advanced epithelialization in the larva-treated groups indicate that the larvae do not merely act as passive “biologically active leeches” but actively stimulate the regenerative phases of healing. This pro-healing activity is likely mediated by a complex mixture of growth factors and cytokines present in larval ES. Studies on *L. sericata* ES have identified components such as transforming growth factor-β (TGF-β) and vascular endothelial growth factor (VEGF), which are critical for angiogenesis, fibroblast proliferation, and epithelial migration (Shanmugam et al., 2022). While the specific composition of *M. domestica* ES is less defined, our histological findings strongly suggest the presence of similar growth-stimulating factors. The robust granulation tissue observed in the BM and BMA groups provides a fertile matrix for healing, underscoring that the therapeutic benefit of MDT extends far beyond simple debridement to active modulation of the wound microenvironment.

### 4.5 Practical implications and future directions

The implications of these findings are substantial for global wound care, especially in the context of rising antimicrobial resistance (Puri et al., 2025). For regions with limited access to advanced wound care products or sterile *L. sericata*, the ability to establish a local, low-cost *M. domestica* bio-rearing program could democratize access to effective MDT. This aligns with the growing push for sustainable and innovative biotherapies (Simonetti et al., 2025). However, the translation of *M. domestica* into clinical practice must be approached cautiously. Future research must prioritize the comprehensive molecular characterization of its ES to fully understand its antimicrobial and immunomodulatory profiles. Rigorous clinical trials in human patients are the essential next step to validate the safety, efficacy, and optimal application protocols for *M. domestica* MDT, addressing potential challenges such as patient acceptance and regulatory standards (Mumford and Nigam, 2024). In conclusion, while *L. sericata* remains the more potent and characterized species, this study firmly positions *Musca domestica* as a scientifically valid, practical, and promising alternative, opening a new avenue for accessible maggot therapy worldwide.

## 5 Conclusion

In conclusion, this study successfully demonstrates that *Musca domestica* larvae are a effective agent for Maggot Debridement Therapy. While *Lucilia sericata* remains the more potent species, the ubiquity, ease of rearing, and proven efficacy of *M. domestica* make it a highly valuable and accessible alternative, particularly in resource-limited healthcare settings where *L. sericata* is unavailable. The combination of MDT with conventional antibiotics presents a highly effective strategy for managing complex, infected wounds and combating antibiotic resistance. Future research should focus on isolating and characterizing the specific antimicrobial and growth-stimulating factors in *M. domestica* secretions and conducting clinical trials to validate its use in human patients.

## Authors' contribution

KA and VS designed and conceptualized the study. QA, SD, and MK gathered the data and analyzed them. SD and MK drafted the manuscript. All the authors participated in writing the manuscript. All the authors have read and approved the final manuscript**.**

## Clinical trial number

Not applicable.

## CRediT authorship contribution statement

**Kourosh Azizi:** Writing – review & editing, Project administration, Conceptualization. **Vahid Saleh:** Writing – review & editing, Investigation, Conceptualization. **Qasem Asgari:** Writing – review & editing, Methodology, Investigation. **Akbar Safaei:** Writing – review & editing, Validation. **Sorna Dabaghmanesh:** Writing – review & editing, Writing – original draft, Methodology, Investigation. **Mohsen Kalantari:** Writing – review & editing, Writing – original draft, Visualization, Methodology.

## Ethical approval

Ethical approval was conducted in accordance with international, national, and institutional ethical guidelines. We declare that all experiments were performed in accordance with the ARRIVE guidelines 2.0 and that all experimental protocols were approved by Ethical approval obtained from the Science and Ethics Committee of Shiraz University of Medical Sciences.

## Funding

None.

## Declaration of competing interest

The authors declare that they have no known competing financial interests or personal relationships that could have appeared to influence the work reported in this paper.

## Data Availability

Available upon request to M.K.
